# Correction: Samadi et al. Autism Spectrum Disorder Diagnostic Criteria Changes and Impacts on the Diagnostic Scales-Utility of the 2nd and 3rd Versions of the Gilliam Autism Rating Scale (GARS). *Brain Sci.* 2022, *12*, 537

**DOI:** 10.3390/brainsci13071081

**Published:** 2023-07-17

**Authors:** Sayyed Ali Samadi, Cemal A. Biçak, Hana Noori, Barez Abdalla, Amir Abdullah, Lizan Ahmed

**Affiliations:** 1 Institute of Nursing and Health Research, Ulster University, Newtownabbey BT37 0QB, Northern Ireland, UK; 2 Bahoz Centre for Children with Developmental Disabilities, Erbil 44002, The Kurdistan Region of Iraq, Iraq; cemal.a.bicak@bahozcenter.com (C.A.B.); hanan@bahozcenter.com (H.N.); barez@bahozcenter.com (B.A.); amir@bahozcenter.com (A.A.); lezan@bahozcenter.com (L.A.)

## 1. Error in Figure

In the original publication [1], there was a mistake in “Figure 3. Comparisons of the number of children in each level of diagnosis of ASD (based on the 3rd version) and the probability of ASD (based on 2nd version of the scale)” as published. Out of 57 children needs to change to out of 52, and 47 (90%) needs to change to 41 (79%). The corrected Figure 3 appears below.

## 2. Figure Legend

In the original publication, there was a mistake in the legend for Figure 1. The corrected legend appears below.

**Figure 1.** Individuals who passed the cut-off score using GARS-2 and GARS-3 scales in the present study.

## 3. Text Correction

1. A correction has been made to Section 1. Paragraph Number 5:

Most ASD instruments are developed in Western societies and cross-cultural psychometric studies are needed from other cultures/societies [21].

2. A correction has been made to Section 1. Paragraph Number 7:

The scales and instruments are generated based on new classifications or in the light of study findings. Some examples are the change in The Autism Diagnostic Observation Schedule (ADOS g to ADOS-2) [25,26], the Childhood Autism Rating Scale (CARS to CARS 2) [27,28], Autism Diagnostic Interview (ADI to ADI-R) [29,30], or different versions of the Gilliam Autism Rating Scale (GARS to GARS-2 and GARS-3) [31–33].

3. A correction has been made to Section 1. Paragraph Number 9:

While Gilliam [31] reported excellent measurement properties, Lecavalier [35] reported lower levels of reliability and validity

4. A correction has been made to Section 1. Paragraph Number 11:

GARS need to be further explored in the “at risk of ASD” population to understand

5. A correction has been made to Section 2.1. Paragraph Number 1:

The area has a young population; 35% of the population is under 15 years of age, and 13% of the households are estimated to have a member with a type of developmental disability.

6. A correction has been made to Section 2.2. Paragraph Number 2:

(Project identification code = BCRD09-2020 approved on 10 December 2020). In the absence of a clear national protocol, this committee adheres to the seventh revised version of WMA of the Helsinki Declaration on Medical Research involving Human Subjects issued on 19 October 2013.

7. A correction has been made to Section 2.3. Paragraph Number 1:

The GARS scale was considered the second level of ASD screening [47,48], and in this study, two final versions of the scale have been used.

8. A correction has been made to Section 2.3.2. Paragraph Number 1:

Using percentile ranks and norms, scaled scores are derived based on each domain’s scores sum, which is used to generate an autism index. An autism index of 54 and lower indicates less likelihood of having ASD, while an autism index between 55 and 70 indicates the probability of

9. A correction has been made to Section 2.4. Paragraph Number 1:

Twelve practitioners undertook the scales in which eight members had a background in psychology (five in clinical psychology, two in special education, and one in educational psychology);

10. A correction has been made to Section 3. Paragraph Number 1:

group comparisons, and tests of significance

11. A correction has been made to Section 3. Paragraph Number 2:

In the present study, data were available for

12. A correction has been made to Section 3. Paragraph Number 2:

screening tool extracted from GARS 2

13. A correction has been made to Section 3. Paragraph Number 3:

Ninety-six individuals (65%) scored about the cut-off based on the GARS-2 and 137 individuals (93%) scored above the cut-off based on the GARS-3 (Figure 1).

14. A correction has been made to Section 3. Paragraph Number 8:

Forty-six cases who did not receive a diagnosis using GARS 2 were diagnosed with GARS-3 (31%).

15. A correction has been made to Section 3. Paragraph Number 9:

all the non-verbal cases “Autism Index” who scored over cut-off with the 2nd and 3rd versions of the GARS

16. A correction has been made to Section 3. Paragraph Number 11:

Correlations between the diagnosis rate and the version of the used scale

17. A correction has been made to Section 3. Paragraph Number 12:

Out of 52 children who, based on the 2nd version, scored under the cut-off score (69), 41 (79%) scored over the cut-off score (55) in the 3rd version and were considered to be at level one of ASD.

18. A correction has been made to Section 4. Paragraph Number 1:

Accessing eligibility to receive ASD services directly depends on the diagnostic criteria; the changing requirements need to be updated or

19. A correction has been made to Section 4. Paragraph Number 1:

The underdiagnosis of ASD cases after the proposed changes to DSM-5 criteria and the scales that became updated based on these changes was a major concern of professionals in the field of developmental disabilities [50].

20. A correction has been made to Section 4. Paragraph Number 2:

Using GARS in this study reported a much higher level of overlap: a rate of 100%. Out of 35.1% (52 children) who scored under the cut-off sore (69) in GARS-2, 79% (41 children) scored over the cut-off sore (55) in GARS-3 and were considered to be at level one of ASD. This was incongruent with the previous prediction that the concern was to exclude cases with higher IQ and those with high-functioning autism or Asperger-like presentations [54].

21. A correction has been made to Section 4. Paragraph Number 3:

To understand and examine the number of individuals diagnosed using these criteria and older and updated ASD criteria, some reviews and analyses have been carried out on the sensitivity and specificity of the DSM-IV-(the 2nd version of GARS has been developed based on the ASD criteria presented in this version)

22. A correction has been made to Section 4. Paragraph Number 4:

Considering that the effect size values ranged from moderate to large, it is indicative that higher scores are more characteristic of severe forms of ASD (levels 3 and 2 based on DSM 5) than those individuals who show milder forms of ASD or ID.

23. A correction has been made to Section 4.1. Paragraph Number 1:

However, the effects of parental attitudes after the diagnosis still needs to be considered in the analysis. The fourth limitation is that there is no confirmed diagnosis of ASD and ID, or the presence of an accompanying diagnostic condition based on gold standard tests or robust clinical examination in the present study.

24. A correction has been made to Section 4.1. Paragraph Number 4:

One of the justifications for the present finding might be due to the cultural aspects and different expectations of the cultural groups.

25. A correction has been made to Section 5:

The more analytical changes are presented for ASD

The authors state that the scientific conclusions are unaffected. This correction was approved by the Academic Editor. The original publication has also been updated.

## Figures and Tables

**Figure 3:**
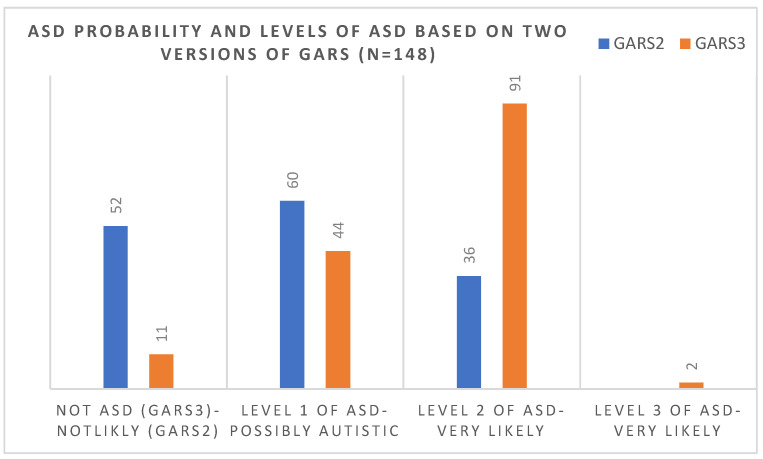
Comparisons of the number of children in each level of diagnosis of ASD (based on the 3rd version) and the probability of ASD (based on the 2nd version of the scale).
